# Structural brain network connectivity in trichotillomania (hair-pulling disorder)

**DOI:** 10.1007/s11682-023-00767-5

**Published:** 2023-04-15

**Authors:** Annerine Roos, Jean-Paul Fouche, Dan J Stein, Christine Lochner

**Affiliations:** 1 Department of Psychiatry and Neuroscience Institute, University of Cape Town, Cape Town, South Africa; 2 SAMRC Unit on Risk and Resilience in Mental Disorders, Department of Psychiatry, Stellenbosch University, Cape Town, South Africa; 3 Department of Psychiatry, Stellenbosch University, Cape Town, South Africa

**Keywords:** Structural network connectivity, Graph theoretical analysis, Trichotillomania, Obsessive-compulsive related disorders

## Abstract

Neuroimaging studies suggest involvement of frontal, striatal, limbic and cerebellar regions in trichotillomania, an obsessive-compulsive related disorder. However, findings regarding the underlying neural circuitry remains limited and inconsistent. Graph theoretical analysis offers a way to identify structural brain networks in trichotillomania. T1-weighted MRI scans were acquired in adult females with trichotillomania (n = 23) and healthy controls (n = 16). Graph theoretical analysis was used to investigate structural networks as derived from cortical thickness and volumetric FreeSurfer output. Hubs, brain regions with highest connectivity in the global network, were identified, and group differences were determined. Regions with highest connectivity on a regional level were also determined. There were no differences in small-worldness or other network measures between groups. Hubs in the global network of trichotillomania patients included temporal, parietal, and occipital regions (at 2SD above mean network connectivity), as well as frontal and striatal regions (at 1SD above mean network connectivity). In contrast, in healthy controls hubs at 2SD represented different frontal, parietal and temporal regions, while at 1SD hubs were widespread. The inferior temporal gyrus, involved in object recognition as part of the ventral visual pathway, had significantly higher connectivity on a global and regional level in trichotillomania. The study included women only and sample size was limited. This study adds to the trichotillomania literature on structural brain network connectivity. Our study findings are consistent with previous studies that have implicated somatosensory, sensorimotor and frontal-striatal circuitry in trichotillomania, and partially overlap with structural connectivity findings in obsessive-compulsive disorder.

## Introduction

Trichotillomania (TTM, or hair-pulling disorder), one of the obsessive-compulsive and related disorders (OCRDs) in DSM-5, is associated with repetitive hair-pulling resulting in hair loss and repeated attempts to decrease or stop the behaviour. The condition is associated with significant distress and impairment in several life domains (American Psychiatric Association, 2013). In a large recent study, point prevalence of trichotillomania in the US was reported to be 1.7% with a lifetime prevalence estimate of 2.5%. Common comorbidities in TTM include obsessive-compulsive disorder (OCD) (Grant et al., 2020). Neuropsychological deficits in TTM include those in response inhibition, working memory, visual memory, and divided attention (Slikboer et al., 2018).

Neuroimaging studies suggest involvement of frontal, striatal, limbic, and cerebellar regions in TTM (Chamberlain et al., 2009; Stein et al., 1997; van den Heuvel et al., 2010). Yet, evidence about underlying neural networks remains limited (Grant, 2019). To our knowledge, there is only one published brain network connectivity study in TTM to date. This study assessed functional connectivity of reward circuitry using resting state MRI, suggesting lower connectivity in frontal, striatal, and limbic regions involved in reward processing (White et al., 2013). The microstructural integrity of white matter tracts has been investigated in TTM in two studies that used diffusion tensor imaging. The one study suggested altered white matter integrity in the anterior cingulate, temporal, and pre-supplementary motor regions (Chamberlain et al., 2010). The other study reported no differences in white matter integrity between TTM patients and controls, but found that white matter integrity in the frontal-striatal-thalamic pathway was associated with illness duration and severity (Roos et al., 2013). Findings from these few studies have thus not always been consistent, emphasizing the need for further study to investigate underlying neural circuitry in TTM.

The global arrangement of a brain network represents a “small-world” organization that optimizes local and global information processing. The changes in structural (and functional) connectivity that have been reported in several neuropsychiatric disorders may be associated with altered topology including in small-world organization, and in the balance between the integration and segregation of information (Bassett & Bullmore, 2009; Fornito et al., 2015; Lord et al., 2017; Menon, 2011). The aim of this study was to derive structural brain networks and investigate its topology in TTM using graph theoretical analysis.

## Methods

### Study design

Participants were recruited by means of general media, e.g., newspaper and online advertisements, and referrals by psychologists and psychiatrists. Individuals who showed interest in the study were screened telephonically and invited to attend comprehensive clinical assessment and brain imaging sessions as part of the study. Patients with a history of neurological illness, psychosis, substance or alcohol use disorder, head trauma, clinically significant depression, or a contraindication to MRI, were excluded from participation. Current daily psychotropic medication use within the last 12 months was also an exclusion criterium. Healthy controls had no current or lifetime history of any DSM-IV disorder, and were without significant neurological conditions and current or lifetime daily use of psychotropic medication. The study was approved by the Health Research Ethics Committees of Stellenbosch University (HREC Ref. M07/05/019) and the University of Cape Town (HREC Ref. 261/2007). Participants provided written informed consent. The study was conducted according to the ethical guidelines of the Declaration of Helsinki.

### Clinical assessments

The Structured Clinical Interview for Obsessive-Compulsive Spectrum Disorders (SCID-OCSD) (du Toit et al., 2001) was updated to include DSM-5 criteria, and used to confirm a diagnosis of TTM. The Mini International Neuropsychiatric Interview Plus (MINI Plus v5) (Sheehan et al., 1998) was used to assess comorbidity. The severity of TTM symptoms was assessed using the Massachusetts General Hospital Hair-pulling Scale (MGH-HPS) (Keuthen et al., 1995). For inclusion in the study, TTM participants had to present with significant hair-pulling, i.e., both in terms of the frequency of hair-pulling, the inability to control the behavior, and the resulting distress. The Clinical Global Impressions Scale - Severity (CGI-S) (Busner & Targum, 2007) was used to assess current global functioning in cases.

### MRI procedures and analyses

Participants underwent structural MRI using a 3-T Siemens scanner to acquire high-resolution 3D-MPRAGE images. Scan parameters were: slab orientation, sagittal; TR/TE, 2300/3.93 ms; flip angle, 12°; FOV, 256 × 240 × 160 mm^3^; and voxel size, 1.3 × 1.0 × 1.0 mm^3^. Cortical thickness and subcortical volume estimates of 86 bilateral brain regions as determined by FreeSurfer v6 (Desikan et al., 2006; Fischl et al., 2004; Fischl & Dale, 2000), were used to construct structural brain networks using graph theoretical analysis (Hosseini et al., 2012; Rubinov & Sporns, 2010). Volumetric data were corrected for individual intracranial volume. The Graph Analyses Toolbox (Hosseini et al., 2012) was used to construct brain networks and determine group differences in brain network organization, while the Brain Connectivity Toolbox (Rubinov & Sporns, 2010) was used to quantify network measures.

Structural networks were created following the steps as described by Hosseini and colleagues (Hosseini et al., 2012, 2013). FreeSurfer morphometric data were corrected for age using linear regression analysis given the range of 18 to 61 years. Residuals from this analysis were utilized to create structural correlation networks. An 84 × 84 association matrix R was made for each group, and every node for a participant denoted a Pearson correlation between residuals of regions i and j (Bernhardt et al., 2011). A binary adjacency matrix A was then created from each R matrix with values set at 1 or 0. Negative correlation values were replaced by zero (Bernhardt et al., 2011; Fan et al., 2011). This provided a binary undirected graph with 86 nodes and a network density that is the fraction of connections to every potential connection.

Metrics defining small-world properties including the clustering coefficient and characteristic path length were determined (Hosseini et al., 2013; Rubinov & Sporns, 2010). The clustering coefficient denotes the mean number of connections of a region with nearby regions, while the mean clustering coefficient signifies network segregation. The characteristic path length denotes the mean shortest path length among pairs of regions that signifies network integration. These metrics are compared to random networks with similar connectivity and distribution of regions (nodes) and connections (edges), to quantify the brain network arrangement (Maslov & Sneppen, 2002; Milo et al., 2002). Such a network has a small-world index >1, including a clustering coefficient that has a ratio > 1 compared to random networks, and a characteristic path length with a ratio close to 1 that is like that of random networks. Other metrics tested for group differences were modularity and transitivity assessing network segregation into communities of regions or clusters respectively, and global efficiency as an indicator of network integration (Fornito et al., 2016; Vertes & Bullmore, 2015).

Regional network connectivity was derived by nodal betweenness centrality, i.e. a measure of regions with highest local connectivity, that is determined as the fraction of all shortest path lengths crossing a specific region (Hosseini et al., 2013). This metric also detects global network connectivity of regions, i.e., hubs that are most connected in the network. A region represents a hub when its nodal betweenness centrality is 1 to 2 standard deviations (SDs) above that of the mean network connectivity (Bernhardt et al., 2011).

Nonparametric permutation tests with 1000 permutations were used to determine group differences in network measures (He et al., 2008). As described by Hosseini and colleagues (Hosseini et al., 2012) in this analysis, residuals of individual participants were randomly re-allocated to either the TTM or control group while retaining the initial sample size per group. An association matrix was derived for every newly randomized group, followed by creation of binary adjacency matrices and determination of network measures at minimum network density. Thereafter, differences between randomized groups in each network measure were determined, creating a permutation distribution of difference below the null hypothesis. The real difference in network measures between TTM and controls were mapped in the relevant permutation distribution and a two-tailed p value determined according to its percentile position. The permutation tests inherently apply correction for multiple comparisons in assessing global metrics using maximal statistics (Nichols & Holmes, 2002; *NISOx: SnPM*, n.d.). Regional group results were corrected using false discovery rate (FDR) (Hosseini et al., 2012). Group differences in brain network parameters were identified at minimum network density (Bernhardt et al., 2011; He et al., 2008) and presented across a range of densities. A brain network has a specific density interval where connections are least random, and every node is connected to at least one other node (Kaiser & Hilgetag, 2006). The minimum density of networks for TTM and controls was 0.24, thus representing the lower bound where networks were not fragmented. The maximum density was 0.44 (small-world index <1.5) above which networks became increasingly random, thus connections would probably not refer to biologically relevant networks above this density (Kaiser & Hilgetag, 2006).

## Results

Demographic and clinical information of participants is shown in Table 1. The sample included adult participants with TTM (n = 23) and controls (n = 16). Our cohort included women only. Age, level of education, and type of employment were similar between groups. Illness severity was generally mild to moderate in the TTM group (mean CGI-S severity score [SD] = 3.76 [1.37]).

**Table 1:** Demographic and clinical information of participants

	TTM	Control	p
	mean (SD) / n, %		
Age (years)	36.09 (14.52)	30.69 (8.19)	0.188
Educational level (n)			0.612
School	9, 40%	5, 31%	
Tertiary	14, 60%	11, 69%	
Employment			0.194
Employed (paid)	13, 56%	12, 75%	
Homemaker (unpaid)	5, 22%	0, 0%	
Student/Scholar	5, 22%	4, 25%	
Ethnicity			0.561
Caucasian	21, 91%	14, 88%	
Mixed Race/Black/Other	2, 9%	2, 12%	
Age of illness onset (years)	13.83 (8.04)	–	
Illness duration (years)	22.26 (16.05)	–	
MGH-HPS total	14.52 (6.26)	–	
CGI-S total	3.76 (1.37)	1.00 (0.0)	

Overall, the brain networks of groups were similar in network arrangement, thus adhering to specified small-world parameters (normalized small-world index p = 0.69) There were no group differences in network measures of segregation (normalized characteristic path length p = 0.37, modularity p = 0.60) or integration (normalized clustering coefficient p = 0.97, global efficiency p = 0.53).

**Fig. 1:**
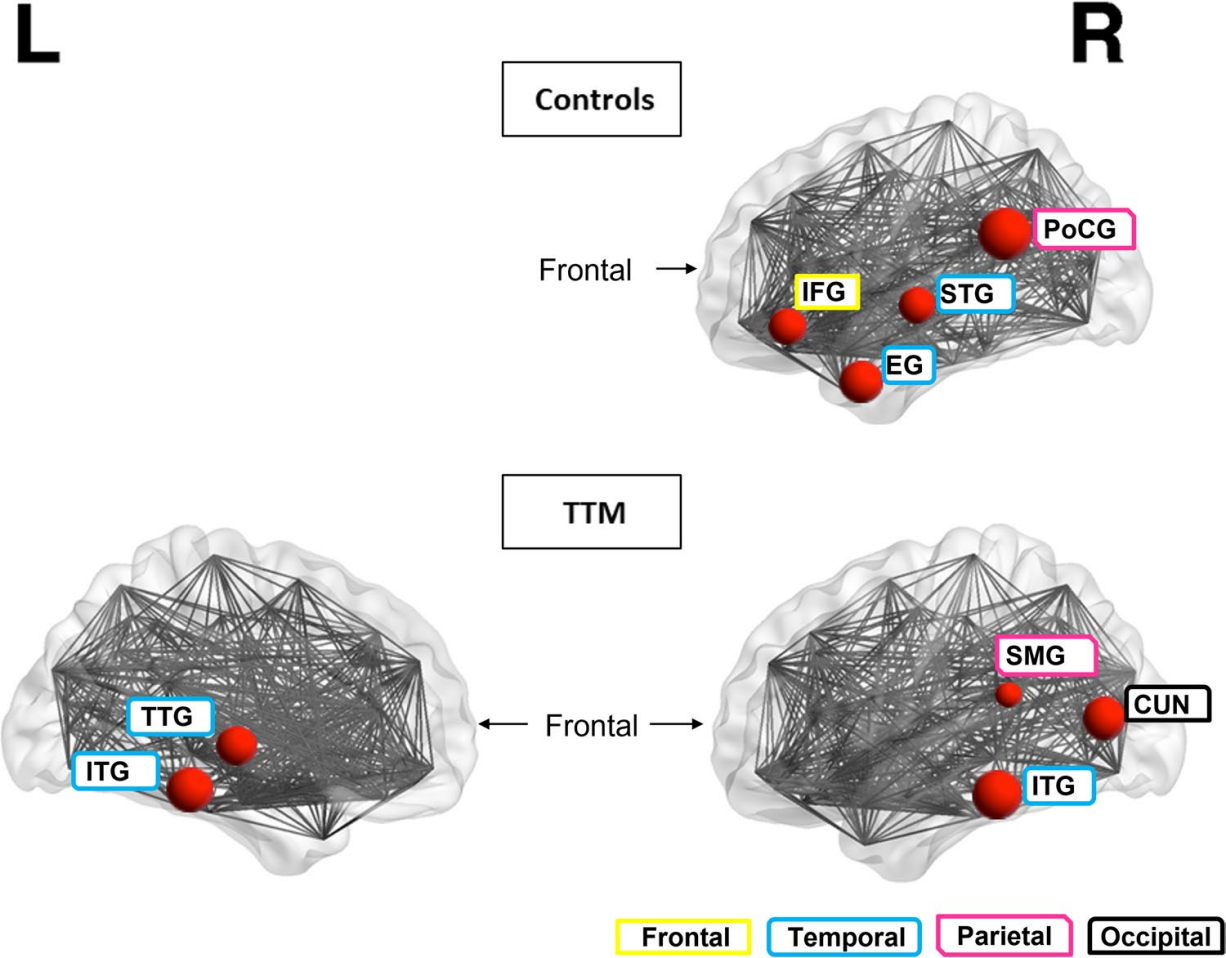
Hub regions at 2 SD within the healthy control [top row] and the TTM group [bottom row]. The hubs in the TTM group were the right (R) supramarginal gyrus (SMG), bilateral inferior temporal gyrus (ITG), left (L) transverse temporal gyrus (TTG) and R cuneus (CUN). The hubs in the control group were the R pars opercularis part of the inferior frontal gyrus (IFG), R posterior cingulate gyrus (PoCG), R superior temporal gyrus (STG) and R entorhinal gyrus (EG). The size of the red circle indicates the number of connections that a hub has in the network. The lobe is indicated at the bottom right by shape- and color-coded legends

Regarding global connectivity, in the TTM group hubs at 2SD were the left inferior temporal gyrus, parietal, and occipital regions, while hubs at 1SD additionally were in frontal and striatal regions (Table 2, Fig. 1). In contrast, in healthy controls hubs at 2SD were in different frontal, parietal and temporal regions compared to TTM. In healthy controls at 1SD hubs were widespread and generally in different regions, for instance, in other segments of the inferior frontal gyrus and the striatum.

**Table 2:** Hubs of the TTM and control groups. Hubs are presented as regions with connectivity of 2 and 1 standard deviation (SD), respectively, above that of mean network connectivity. Hubs identified at both SD levels per group are indicated in italics, primarily representing parietal, temporal and occipital regions in TTM. Frontal and striatal hubs were evident in the TTM group at 1 SD above that of mean network connectivity

Lobe	TTM	Control	TTM	Control
	2 SD	2 SD	1 SD	1 SD
Frontal		*R Pars opercularis*	L Pars orbitalis	*R Pars opercularis*
			R Pars orbitalis	L Pars triangularis
			L Lateral orbitofrontal	L Frontal pole
			R Lateral orbitofrontal	
			R Caudal anterior cingulate	
Parietal	*L Postcentral*	*R Posterior cingulate*	*L Postcentral*	*R Posterior cingulate*
	*R Supramarginal*		*R Supramarginal*	L Supramarginal
				L Postcentral
				R Postcentral
				R Paracentral
Temporal/limbic/striatal	L Inferior temporal*	*R Superior temporal*	L Inferior temporal*	*R Superior temporal*
	*R Inferior temporal*	*R Entorhinal*	*R Inferior temporal*	*R Entorhinal*
	*L Transverse temporal*		*L Transverse temporal*	R Banks superior temporal sulcus
				R Isthmus of cingulate gyrus
				R Parahippocampal
			L Caudate	R Ventral diencephalon
Occipital	*R Cuneus*		*R Cuneus*	R Precuneus

*Highly connected both at a global and regional level

Regarding regional network connectivity, there were significant differences between study groups in two regions. Local connectivity was significantly higher in TTM compared to controls in the left inferior temporal gyrus (p < 0.001). Local connectivity was significantly higher in controls compared to TTM in the right pericalcarine gyrus (p = 0.04), located below the cuneus in the occipital lobe.

## Discussion

To our knowledge, this study is the first to use graph theoretical analysis to investigate structural brain networks in TTM. The main findings suggest 1) similar small-world properties and network measures including segregation and integration between patients and controls, 2) involvement of different hubs in the global network of TTM patients compared to controls, including inferior temporal, parietal and occipital hubs (at 2SD above mean network connectivity) and frontal and striatal hubs (at 1SD above mean network connectivity), and 3) a role for the inferior temporal gyrus on a global and regional level in TTM, involved in object recognition as part of the ventral visual pathway.

Corresponding small-worldness and network measures in both TTM and controls suggest brain networks that generally have similar network arrangement. This is partly consistent with some studies in OCD, the key example of the obsessive-compulsive and related disorders (OCRDs), that found similar small-world properties compared to controls (Kim et al., 2013; Reess et al., 2016). Earlier studies suggest involvement of cortical-striatal networks in both TTM and OCD (Chamberlain et al., 2009; de Wit et al., 2014; van den Heuvel et al., 2010). Potentially relevant is recent evidence in OCD that shows distributed involvement with altered connectivity in cortical-striatal-thalamic-cortical networks, the default mode network, in specific frontal, parietal, temporal and limbic regions within networks, and the cerebellum (Hou et al., 2013; Kim et al., 2013; Reess et al., 2016; Zhong et al., 2014).

Our findings indeed suggest a distributed set of regions operating as hubs in the global network in TTM. These include temporal, parietal, and occipital regions, as well as frontal and striatal regions. Hubs found across lobes in the TTM group broadly coincide with regions implicated in TTM using other imaging modalities, e.g. diffusion tensor imaging and resting state functional imaging (Slikboer et al., 2018). Notably, the inferior temporal gyrus had significantly high connectivity on a global and regional level. This gyrus has a role in object recognition as part of the ventral visual pathway (Conway, 2018; Kanwisher, 2010). Aberrant connectivity of the temporal and parietal hubs located in somatosensory and sensorimotor networks also likely underlie core symptomatology in TTM, including over-responsivity to external sensations in tactile and auditory domains (Falkenstein et al., 2018). This provides support for earlier findings suggesting involvement of the inferior and superior parietal cortex and left somatosensory cortices (Chamberlain et al., 2009, 2010; Swedo et al., 1991), and the left temporal lobe (Chamberlain et al., 2010; Odlaug et al., 2014; Warrier et al., 2009) in TTM. Regarding the cuneus of the occipital lobe, this hub functionally forms part of the primary visual and somatosensory networks (Tomasi & Volkow, 2011). Cortical thickness of the right cuneus has been associated with impulsivity (Kubera et al., 2018) and sensation seeking (Miglin et al., 2019) which, along with novelty-seeking, have been associated with TTM (Flessner et al., 2012). Higher volume of the right cuneus and superior occipital lobe where this region is located, has also previously been reported in TTM (Chamberlain et al., 2009; Grachev, 1997), and may suggest increased neural connections that affects its operation within networks. Altered structural connectivity of occipital lobe regions in visual and sensory networks may contribute to altered attention to and perception of spatial detail, and aberrant behavioral control in TTM.

The current study findings also suggest involvement of frontal-striatal circuitry in TTM. Consistent with aspects of earlier studies (Grachev, 1997; Odlaug et al., 2014), we found that anterior and inferior frontal regions were highly connected in the global network of the TTM group. Our hubs represented the lower segment of the inferior frontal gyrus, i.e. bilateral pars orbitalis, which is located adjacent to the lateral orbitofrontal cortex that was also a hub in our TTM group, and also structurally altered in OCD in previous studies (Fouche et al., 2017; Venkatasubramanian et al., 2012). Finally, our finding that the left caudate is a hub in TTM supports earlier work suggesting an association between symptom severity of TTM and left caudate activity (Stein et al., 2002). Of note is that structural abnormalities have also been reported in other parts of the dorsal striatum in TTM, including the right caudate (Isobe et al., 2018) and left putamen (Chamberlain et al., 2009; O’Sullivan et al., 1997). The dorsal striatum is involved in decision-making particularly about actions, reward, and habit formation resulting in automated behaviors based on sensorimotor, cognitive, affective and motivational determinants (Balleine et al., 2007; Lipton et al., 2019). Thus, although the evidence is limited, and somewhat mixed, it appears that the dorsal striatum may have altered structural connectivity and function in TTM.

Hubs in our controls represented principal regions typically found in structural networks of healthy adults, which include those of the default mode network (Hagmann et al. 2008; van den Heuvel & Sporns, 2013). On a regional level, the pericalcarine gyrus situated in the occipital lobe that operates within the greater sensorimotor network, had lower connectivity in TTM patients than controls. Interestingly, lower pericalcarine cortical density has been associated with sensation seeking behavior (Miglin et al., 2019), a tendency that may be relevant to TTM (Lejoyeux et al., 1998).

This study had limitations. First, the study included women only, in line with epidemiological data suggesting that the majority of individuals with TTM are female (American Psychiatric Association, 2013; Lochner et al., 2010; Woods et al., 2006). Second, sample size was limited. Nevertheless, with our whole brain explorative approach, this study of structural brain network connectivity in TTM using graph theoretical analysis, is novel. Third, structural networks were created using inter-regional correlations at group level and therefore do not reflect networks at individual level. Therefore, other factors that may determine underlying structural covariance such as clinical measures could not be associated with network measures. Results may also be interpreted differently depending on parcellation strategies, differences in network density, the strength of connections and whether a network is weighted or not (Farahani et al., 2019). Although every network measure provides information on the topology of the brain, cortical thickness represents one of several types of parcellation schemes used to define structural connectivity or covariance.

## Conclusions

Our findings largely coincide with previous studies implicating somatosensory, sensorimotor and frontal-striatal circuitry in TTM. Our findings also partially overlap with structural connectivity findings in OCD, a key example of the OCRDs. Further study in larger samples is needed to differentiate structural and functional networks of this condition.

## Data Availability

Data of this study are available from the authors upon reasonable request as per cohort guidelines.
